# Supplementary material for: A screen for synthetic genetic interactions with the Saccharomyces cerevisiae hrq1ΔN allele
Source: G3 (Bethesda). 2025 Sep 25;15(12):jkaf222. doi: 10.1093/g3journal/jkaf222 (PMC12693483; doi:10.1093/g3journal/jkaf222)
Supplement: jkaf222_Supplementary_Data [file jkaf222_supplementary_data.zip › Supplementary_Figures,_Supplementary_Table_1,_and_Legends_G3-2025-406047.docx]

**SUPPLEMENTAL MATERIALS**

**Supplementary Table 1.** AlphaFold 3 inputs.

| **Protein** | **Sequence** |
| --- | --- |
| *Saccharomyces cerevisiae* Hrq1 | MEEGPIKKKLKSAGQGSGKTDAFRNFEQFFFRLNTLYTFLICRKHVVPTFKTLCGPIETALKRTVTKEDLAMVMALMPRECVFKYIDENQIYTETKIFDFNNGGFQQKENDIFELKDVDDQNQTQKSTQLLIFEFIDGTMQRSWSASDRFSQIKIPTYTTEEMKKMISKREALFKSRLREFILEKEKANLDPFSELTNLAQKYIPRERDYEDPIEAMMKAKQESNEMSIPNYSNNSVITTIPQMIEKLKSTEFYASQIKHCFTIPSRTAKYKGLCFELAPEVYQGMEHENFYSHQADAINSLHQGENVIITTSTSSGKSLIYQLAAIDLLLKDPESTFMYIFPTKALAQDQKRAFKVILSKIPELKNAVVDTYDGDTEPEERAYIRKNARVIFTNPDMIHTSILPNHANWRHFLYHLKLVVVDELHIYKGLFGSHVALVMRRLLRLCHCFYENSGLQFISCSATLKSPVQHMKDMFGINEVTLIHEDGSPTGAKHLVVWNPPILPQHERKRENFIRESAKILVQLILNNVRTIAFCYVRRVCELLMKEVRNIFIETGREDLVTEVMSYRGGYSASDRRKIEREMFHGNLKAVISTNALELGIDIGGLDAVLMCGFPLSMANFHQQSGRAGRRNNDSLTLVVASDSPVDQHYVAHPESLLEVNNFESYQDLVLDFNNILILEGHIQCAAFELPINFERDKQYFTESHLRKICVERLHHNQDGYHASNRFLPWPSKCVSLRGGEEDQFAVVDITNGRNIIIEEIEASRTSFTLYDGGIFIHQGYPYLVKEFNPDERYAKVQRVDVDWVTNQRDFTDVDPQEIELIRSLRNSDVPVYFGKIKTTIIVFGFFKVDKYKRIIDAIETHNPPVIINSKGLWIDMPKYALEICQKKQLNVAGAIHGAQHAIMGMLPRFIVAGVDEIQTECKAPEKEFAERQTKRKRPARLIFYDSKGGKYGSGLCVKAFEHIDDIIESSLRRIEECPCSDGCPDCVAASFCKENSLVLSKPGAQVVLHCILGHSEDSFIDLIKDGPEPNMPEIKVETVIPVSEHVNFSDDFKIIDVRRATKDDTHTNEIIKKEI |
| *Schizosaccharomyces pombe* Hrq1 | MSQTPIKKEESNDQDDKFEFKKYINEGKLPLKADNPKKKPQLGTIQANQPIPSIFDNLFNLFKVINTTYTFLYLRNSLTITFPLLNSSVKQSLKKELTIGDLSQLREICPQIIELNYKSLASLALEINKNVYTDLNPELYTGSTVSQSSEYVLVIELLETQERSSKRRRREGPTMKANIQRQKLDFNNLKKAIELRNQKFLQGIKEYIKKCQLTELDPTQQLLTQSRKNQPVPPDSPSIPNDSIENCNLNTKACSIEELLNEIASESSYEGQIVQEALHTYPAVEAQYGALSRPLSQELINALYTSRNIEKTYKHQADAINHLWNGFHVIVSTSTSSGKSLIYQIPILQSLLEDNQSTAFFVFPTKSLAQDQKKSLIDILSYMPTLKNIRVDTFDGDTPLESRESIIRSANIIFTNPDMLHQTILPNANRWYYFFKNLKLFVLDEAHVYNGIFGVHVAFVLRRMRRIAEYFGNSQYRFVSCSATIEDPLQHMKKIFGVDNIKLINYTSSPSGSKKFVMWNPPYVDPKHPDDGKKSAISEASKLLIKFAEKRVRTIVFCRVRKTCESLMRLVRQELKTKQKGDLLSKIQSYRAGYTVQERRKIESEMFNGKLYGIIATNALELGIDIGSLDAVITIGFPYSLSNLRQQFGRAGRRNKSSLAVYIVETFPVDQFYLKHPILIHTQPNAELTLDLTNEVLLASHLQCAAYELPINIRSDEKFFGNQIQDICEANLEMVEESYRPHPKYLPFPASQVRIRSVSEDMFTLVDVTNDKNVILELLEPFRVALTAYEGAVYVYQGKTFIIRLLNINKRIITAHQVDVEWSTLQRDFTDVDPVRSLMKKTMHGSTNIYFGAVKATLHVFGYFKVNKQKDILDVVDITDHPVEIDSRGFWIDVPWHIIEVLSLKKINGAASIHAAQHALLSLMPIFISNSGNDIRTECKAGEKEYKEAKSERRRPSRLIFYDNCGDSSGAGLCNKAYEHTDELITMAIERIESCDCKVREGCPGCITSSKFEGGVCSGEVLDKVGALILLKMLLCQHVNLDIYADGPEIDSYHALRTLIPSC |
| *Homo sapiens* RECQL4 | MERLRDVRERLQAWERAFRRQRGRRPSQDDVEAAPEETRALYREYRTLKRTTGQAGGGLRSSESLPAAAEEAPEPRCWGPHLNRAATKSPQSTPGRSRQGSVPDYGQRLKANLKGTLQAGPALGRRPWPLGRASSKASTPKPPGTGPVPSFAEKVSDEPPQLPEPQPRPGRLQHLQASLSQRLGSLDPGWLQRCHSEVPDFLGAPKACRPDLGSEESQLLIPGESAVLGPGAGSQGPEASAFQEVSIRVGSPQPSSSGGEKRRWNEEPWESPAQVQQESSQAGPPSEGAGAVAVEEDPPGEPVQAQPPQPCSSPSNPRYHGLSPSSQARAGKAEGTAPLHIFPRLARHDRGNYVRLNMKQKHYVRGRALRSRLLRKQAWKQKWRKKGECFGGGGATVTTKESCFLNEQFDHWAAQCPRPASEEDTDAVGPEPLVPSPQPVPEVPSLDPTVLPLYSLGPSGQLAETPAEVFQALEQLGHQAFRPGQERAVMRILSGISTLLVLPTGAGKSLCYQLPALLYSRRSPCLTLVVSPLLSLMDDQVSGLPPCLKAACIHSGMTRKQRESVLQKIRAAQVHVLMLTPEALVGAGGLPPAAQLPPVAFACIDEAHCLSQWSHNFRPCYLRVCKVLRERMGVHCFLGLTATATRRTASDVAQHLAVAEEPDLHGPAPVPTNLHLSVSMDRDTDQALLTLLQGKRFQNLDSIIIYCNRREDTERIAALLRTCLHAAWVPGSGGRAPKTTAEAYHAGMCSRERRRVQRAFMQGQLRVVVATVAFGMGLDRPDVRAVLHLGLPPSFESYVQAVGRAGRDGQPAHCHLFLQPQGEDLRELRRHVHADSTDFLAVKRLVQRVFPACTCTCTRPPSEQEGAVGGERPVPKYPPQEAEQLSHQAAPGPRRVCMGHERALPIQLTVQALDMPEEAIETLLCYLELHPHHWLELLATTYTHCRLNCPGGPAQLQALAHRCPPLAVCLAQQLPEDPGQGSSSVEFDMVKLVDSMGWELASVRRALCQLQWDHEPRTGVRRGTGVLVEFSELAFHLRSPGDLTAEEKDQICDFLYGRVQARERQALARLRRTFQAFHSVAFPSCGPCLEQQDEERSTRLKDLLGRYFEEEEGQEPGGMEDAQGPEPGQARLQDWEDQVRCDIRQFLSLRPEEKFSSRAVARIFHGIGSPCYPAQVYGQDRRFWRKYLHLSFHALVGLATEELLQVAR |
| *Chaetomium thermophilum* RecQ4 | MPVSSNDGAAPWARTRGGGSLGKRKRGQTADEKGSPSSSQSIDSSGLHKIGSGEATEPLNSSDVASVETRSQETWPQNQTSKHPTTTRRRNQPKAKTKNPAPEEDNSDPSPKSTSTNGKGKTQKPSERSRPEPKPSSWPPYLSRLSKLHRALNLVTTFLAARRASHLTPLALESVKEAVQGQCGFGVEVEDVAGVVWVYAWGEARELEAQGERNEDQEQGGGSNQHNWGRQIQGEGQVKRCSRVRFEYVTQEAEDEFDANAKGEQVLVFEFLELDKQLKPEKEKGRGSKEREDKIRMPSVGAKQLSKVIERREEKFAEMVELFVLSCKQRGKDPDEVVKQGRGRFIPRLPEDTPPNPDSLPLTIPTERKPISEIVEELKCSSWYVGQIVPDGHRVLDPQEAIYGELNFPLGQDLVNALYNSKGITPDRLYSHQAAAINALHAGHHVVVATSTSSGKSLVYQLPVLHVLEEDKNARAMYIFPTKALAQDQKRSLQELISWMPSLEGIVVETYDGDTPQEYRRSIRENASVIFTNPDMLHAGVLPQEEGWRMFLKGLRYVVVDELHYYNGLLGSHVAWVMRRLRRVCAALGNSDVKFVSCSATVANPEGHFRRLFGIEDNVTVIDFDGSPSGRKEFLVWNTPYKVPGDPSSGRGNALLECSRLFCQLILRGVRTIAFCRVREQCEKLVAAVKKELESLDRGEVAGRVMAYRGGYTAQDRRQIEADMFSGRLVGIVATTALELGVDIGTLDCVLTWGFPYTIANLRQQSGRAGRRNKDSLAILVGDAFATDQYYMQHPDELFSRPNCALQIDLDNLLVKEGHLQCAAGEMPIHPEQDKIYFGPDLPQLCAERLIPDDEGFYHCHDHFRPFPWRFVSIRDVEEDKFAIVDTTNGRNVVLEELDASRATFTIYDGAIFLHQGTTYLVRDFSPDRKIARVEKVKVDYLTSQRDFTDVDPIATLAIKPLQGSPYKAYYGTIRITQVVFGFFKVDPRRGNKILDAVPVHNPPVVRYSKGMWLDIPACALDILKARKHNPAAAIHAAQHALASLVPTFVMSSTQADIRTECKSPIKEFARRPTNRKRPARLALYDAKGGPGGAGINTKAFEFVDDLLRMALDRVRGCECLEEKGCVECVCSEFCVEGNEVMSKVGAEVVLKVLVGEEVREEEVPIGSWEEGMGPGETVVLAGEVSMASGSGSQQDGR |
| *Mus musculus* RecQ4 | MERLATVRARLQEWERAFARLHGRRPAKGDVEAAPEETRALYREYRNLKQAVRQADDRHRVLEQSLAEAAEEAQEPSCWGPHLSRAATQNTQSMPKQSLLSSVQDYGKRLKANLKNTTQTGPTQSRKLQLQKRSLSTVPAPRPPGSKTESPCPDEADDALPRVPEPRPRLGQLQQLRSSLSRRLTSLDPGWLERCHNRVSDLLEVPGACGLDLSAEESQPQMSGKVNIADPDIQSEVSVQSPEAIAQQPAQVLSQSPKSINSKGRKRKWNEKGEDFAQDQPSSGAGPLSEGARATVHGQDPPGEPTQVNVPQPCNSSNQARTEKAKGTTHLHASPRPASLDRGNYIRLNMKNKRFVRVGANRGRLLRKQVWKQKWKKKQAAFGGSGPRATDKDTCFRCGQFGHWASQCSQPGPTLTVQEEGDRDDKQPISTLEEVAQRTGTASCHHSGEETQPAAPELQVPHCPTPMSPLYPPGPLGQVAETPAEVFQALERLGYRAFRPGQERAIMRILSGISTLLVLPTGAGKSLCYQLPALLYAQRSPCLTLVVSPLLSLMDDQVSDLPSCLKAACLHSGMTKKQRESVLKKVRAAQVHVLIVSPEALVGCGARGPGSLPQAAQLPPIAFACIDEVHCLSQWSHNFRPCYLRVCKVLREHMGVRCFLGLTATATRSTARDVAQHLGIAGEFELSGSANIPANLHLSVSMDRDSDQALVTLLQGDRFRTLDSVIIYCTRERIQNGWLALLRTCLSMVGDSRPRGCGPEAIAEAYHAGMSSQERRRVQQAFMRGHLRMVVATVAFGMGLDRPDVRAVLHLGLPPSFESYVQAIGRAGRDGKPAHCHLFMHPQGEDLWELRRHAHADSTDFLAVKRLVQRVFPPCTCSQRPVSKSSPEEVKEHSGQQTYPVLGQACLGHERALPVQSTVQALDMTEEAIETLLCYLELHPRHWLELLPWTYAQCHLHCLGGSAQLQALAHRCPPLAACQAKWPPKDTSQGRSSLEFGVVELADSMGWKLASVRQALHQLKWDPEPKKGAAQGTGVLVKFSELAFHLHSRGDLTDEEKDQICDFLYNRVQAREHKALAHLHQMSKAFRSVAFPSCGPCLEQSNEEHSNQVKTLVSYYFEEEEEEEETMTDTQGPKPGQTQLQDWEDQIRRDVRQLLSLRPEERFSGRAVARIFHGIASPCYPAQVYGLDRRFWRKYLHLDFHALMHLATEELLLRGR |
| *Zea mays* RecQ4 | MQANKPPKVNWPHHENAVQGYSSRDDFLSSSFLFSLPTQRPNPEARERMLSLRSSACKIQGPERLQAPLIEKAWRSLCNTQAACKSYLRPGLSAKARDCDRGHARTYGEGSYNTNKMSTVPGNRILSMESTGQPSERVSLQNNSSHQTVGICSSMRSHQNNHVVQEDMRATNQYNFARTNAALHQSMAADNMCSYDKFDAMDDDILATIDVDQIVMEHYQATNAPRGSASHNMSTPPGNKCSVNGMDEANLPRELSELCNHQCKLAFCREAMTHLQEMKDELLAVANELLDDDGELNPQHSQELHKRRLHLKKLVQLLEDHMTRSAQDEERQISHSMASTTSTQQHLPPMTPGSTFTMDSNRFQSQVYVGNGPRDSDLCYSPAPYSCSDNLSTPLNSVWKSYTPKVIDINYTEGSGDRKWSSTNFPWTKELEAKNRNKFGNHSFRPNQREIINATMSGHDVFVLMPTGGGKSLTYQLPALISSGLTLVVCPLVSLIQDQIMHLSQANIPATYLSGNLDWSEQQEIMKDLMSCRYKLLYVTPEKIAKSGALSRLLDNLNSQGHLSRIVIDEAHCVSQWGHDFRPDYKSLGVLKQNFPKTPVLALTATATARVKEDVVQALALENCIVFKQSFNRANLRYYLRPKTKKCVEDIDLFIRTNHSKECGIIYCLSRMDCEKVAEKLRDCGHKVSHYHGSMDPMDRTHIQKLWSKDKINIICATVAFGMGINKPDVRFVIHHSLPKSIEGYHQECGRAGRDGQPSSCVLYYQYSDYIRVRHMITQGVTEQTGAPRDLSSHEQALKTHKDNLLRMVSYCENDVDCRRLLQLIHFGEKFDPSLCARTCDNCLKESGWVEKDVTNIARQLVELVTRTGHSHSSTYILEVYRGSMSQNVKKQRHDALALHGAGRHLAKGEAARIMRHLVTEEILNEDVKKSDMYGSISSVLKVNHLKASGLLSGKHNIVLKFPASDKASKMRNLDASLLPQTNKNVQQQSEVDVKLASMLYEALLSLREQIMEECNEGFNAYHIFKTDTLKEMSIRVPRTKEELLDINGLGKTKVKKYGDRLLATIEDFLSKHPNPRRNSGGGGGGNEHSDAAKKRRGSTAISAASYGDDDFDERTGQQSKKRAAKTRTVSAHGPRCTDADLDGPEVVEVDGELRSVRKPVAYGNGRVLPKWAPL |
| *Drosophila melanogaster* RecQ4 | MYYKLKTSFLEETLNDVLSEDGYDILEMSQASDLGVSMLDQDVSLNEGPQLPLDISALVGPQSSGNLEEIPQSVEGSFSNLIDLPNRQVLTNLVNRDENHVIRKFEAAEELPINQNAWGLNVSKKPPAPPQPVEASKSAPGHGKQPKAGASLKPSLSAKLFQSSRGFAKRNPRKPLSRCVSSSSSTTSLSSVPTDHHEELLDFETILIRKAQEYKEKQQAIANNPMLASEHSKESIKTLVDDGWLRRNTKENTLDEEVPFAEANNNSGTSKKTNFGLANLDLSKLKPTVKEEKVLQAKPDQMAIIQELQTDMNSSMNQKPDHLNHTPPASSQKSVAPKNKPPPSEQETDSDSDSVVAESEEEQEPQEYRQLSKRRKIVSTASGKVEVAAPVEIPNKVEPETETFAQENPDFSADEDQDATYVPENKKKDKAKRKQAAGKQKTTKPKAEPKPKTEKKAKVKAEKKPKAEKKPRNSKKAIAVEPAPDPEEDERQPLNPEDLKYVLALEAGDITSVPRINVQDLEEADATAQRYIRTFAAGPNPGLSEGSNIRVDEKRAAARKKLEERIAAGKLNENFVTINIQKKKFVRGKKSVNFSKYKKQQWRHKKRVAALSGPDMDMGGCDGGVLTCFQCGGVGHFAQQCKVKGDSLLPLSAQLEEDPSPFPTLAEAQEMASQGAVVAHSRNISRLPQAANAAILQGDELNESEEDDQESSEDEEVQQHPDPNWSSDEMDVDFEALDAAVEASLSQPVSQEKAASPIKTYVGHKIPEEFLKQAGLDTTASSSNRSQHGGVKPLYDLLPDGSVQDTTPEVLEALHMFGHTNFRKGQDRAIMRTLSGLSSLVTLSTGSGKSLCYQLPAYLYSRKVGAITLVISPLVSLMEDQVTGVPHFLRAHCLHTNQTAPQRMKIQQMIANGEIDILLVSPEAVVAGERATGFGAILRQLPPIAFACIDEAHCVSQWSHNFRPSYLMICKVLRKNLGVRTVLGLTATATLPTRVSIINHLGISDGERGIISDIPLPDNLVLSVSKDENRDAALLQLLNSERFEPCQSIIIYCTRRDECERIAGFIRTCVQDRREPTQDQTKKRKRVNWQAEPYHAGMPASRRRTVQKAFMSNELRIVVATIAFGMGINKPDIRAVIHYNMPRNFESYVQEIGRAGRDGLPSHCHLFLDAKGGDQSELRRHVYSNSIDRHVIRKLLQKIFVPCSCDKEASKRTALPIPLEGDGPRVHMCPGHEIGFSVEKTVEMLDIPAENISTLLCYMELDPRWCISVLSSAYVMAKVISYGGPKYLKHAAKECPPLAMAIALQIRDKTFKEDSNIIEFSVTDIAAGIGWNSGVVKYQLKDLEWVKVNGYPKRSPITVSFYDLGFRIKVPGDFTESEIDNALDTLYTRSVKQERTQLIQLQYVAHGLAAVAYSSCGQCCNADFPQDRGEQLKAIVRNYFANDYPQDLELEIEPSNVPDENIIDDVHALINMYPDNTFTGRNIARIFHGIMSPNYPAVIWGRCKFWRAHVKVDFNRILHLANMAIIKRRT |

**Supplementary Table 2.** *hrq1ΔN* SGA data (see file S2).

**Supplementary Table 3.** Shared and unique positive synthetic genetic interactions between *hrq1Δ* and *hrq1ΔN* or *hrq1-K318A* and *hrq1ΔN* (see file S3).

**Supplementary Table 4.** Shared and unique synthetic genetic interactions between *hrq1-K318A* and *hrq1ΔN* (see file S3).

**SUPPLEMENTAL FIGURES**

**A)** ScHrq1 (*Saccharomyces cerevisiae*): N-terminus (aa 1-279)


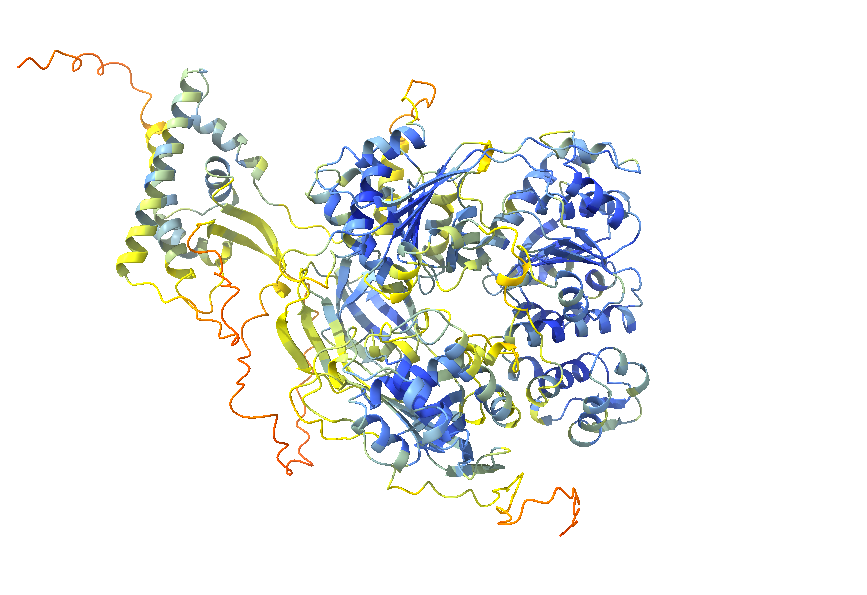

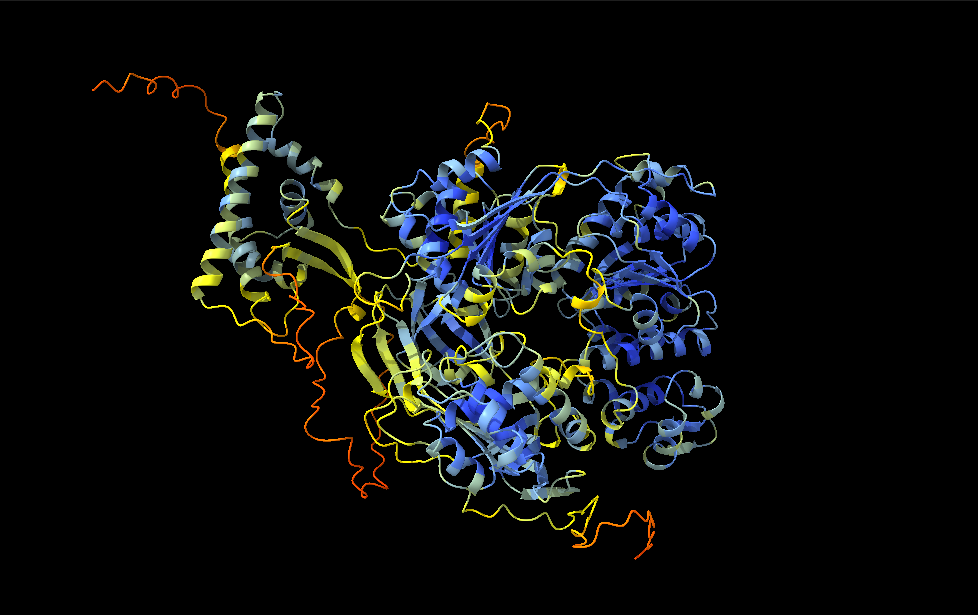

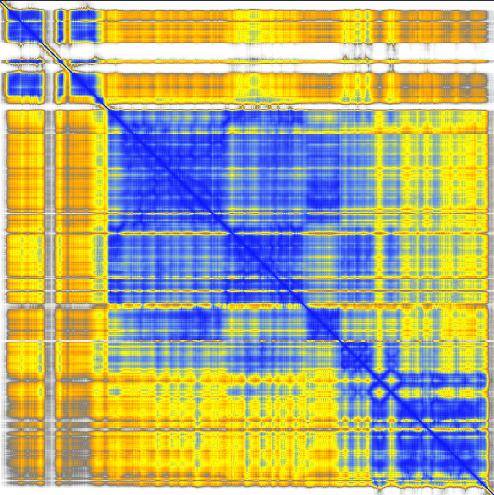


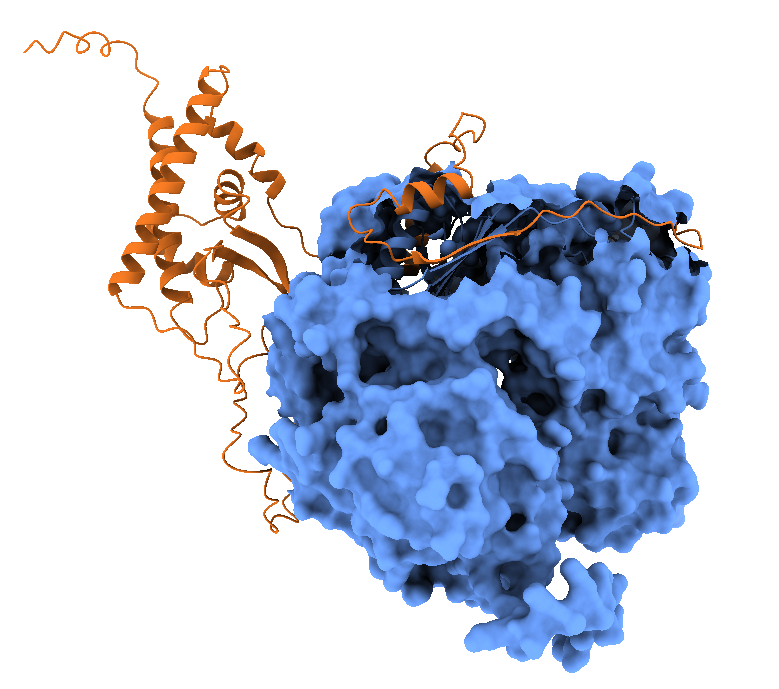

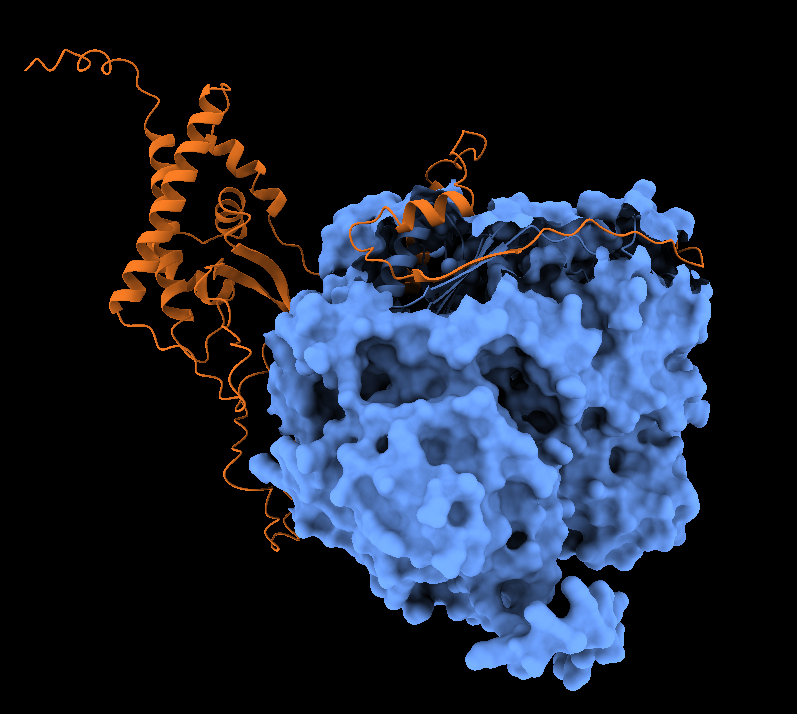


**B)** SpHrq1 (*Schizosaccharomyces pombe*): N-terminus (aa 1-255)


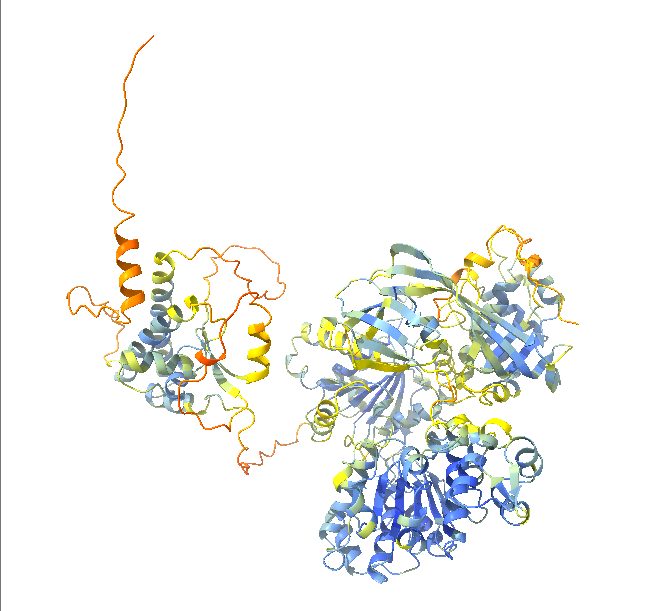

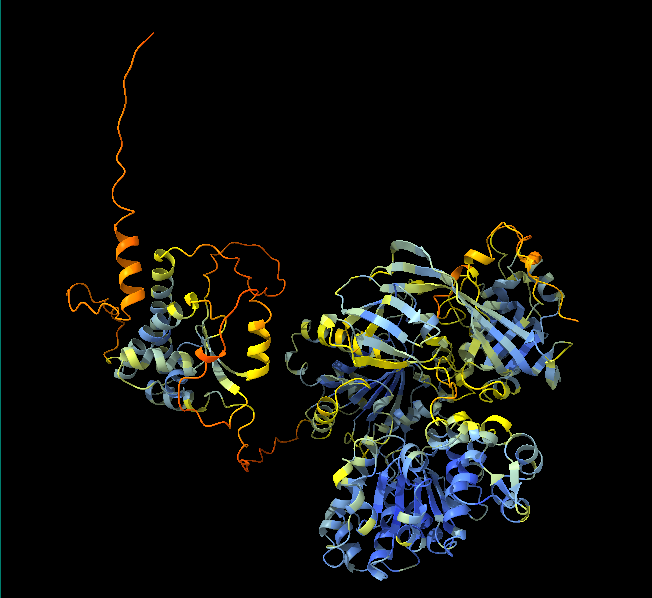

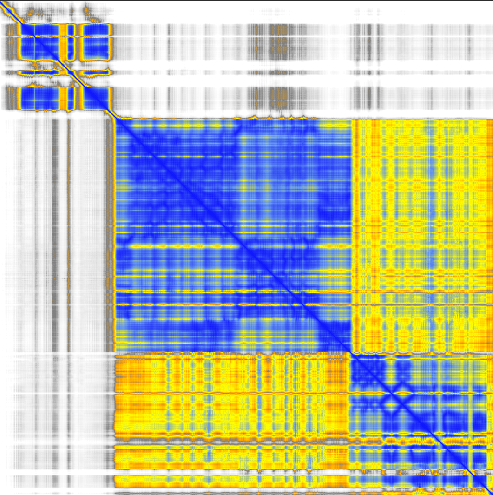


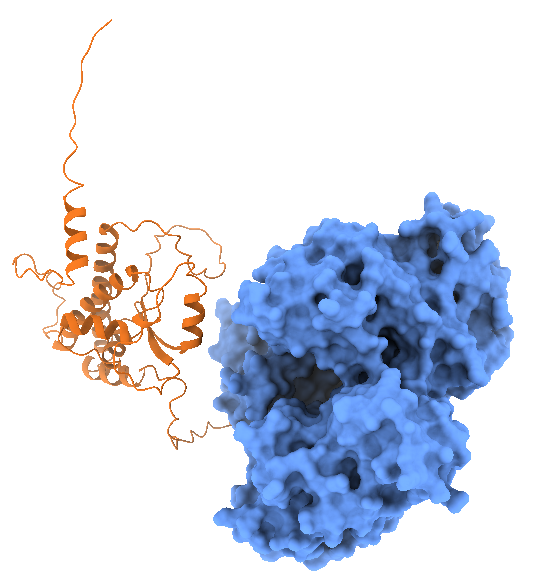

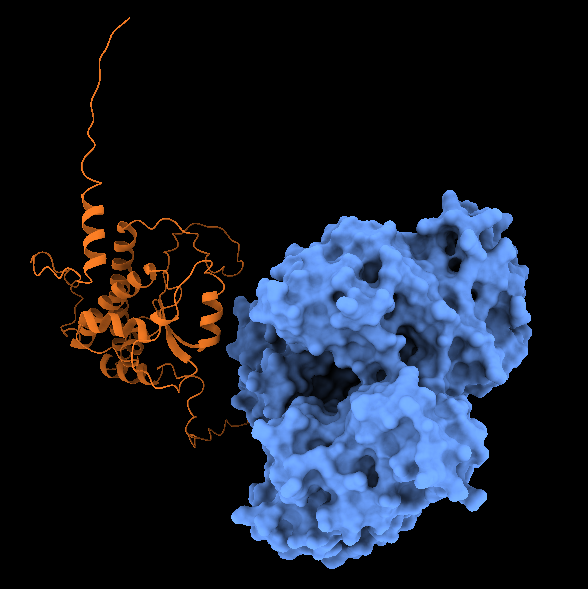


**C)** hRecQ4 (*Homo sapiens*): N-terminus (aa 1-475)


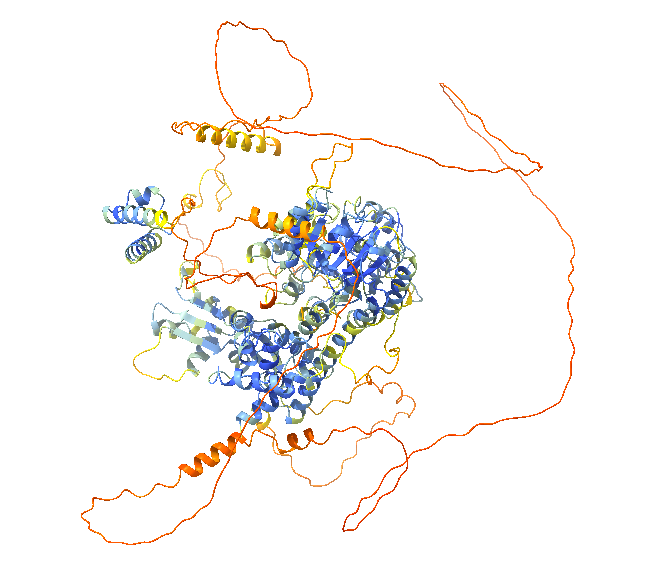

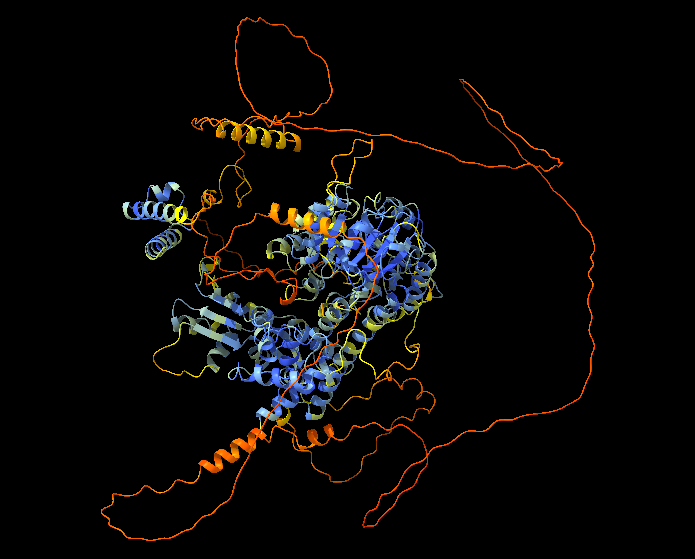

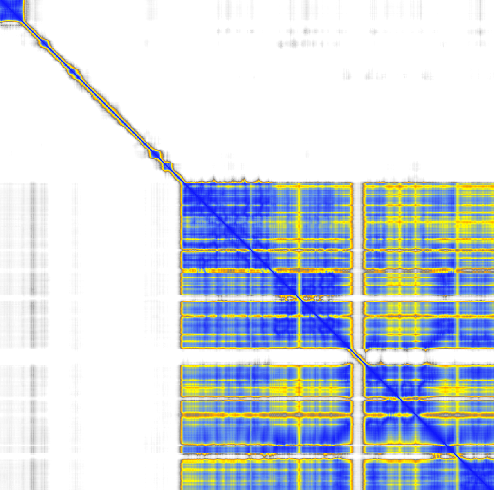


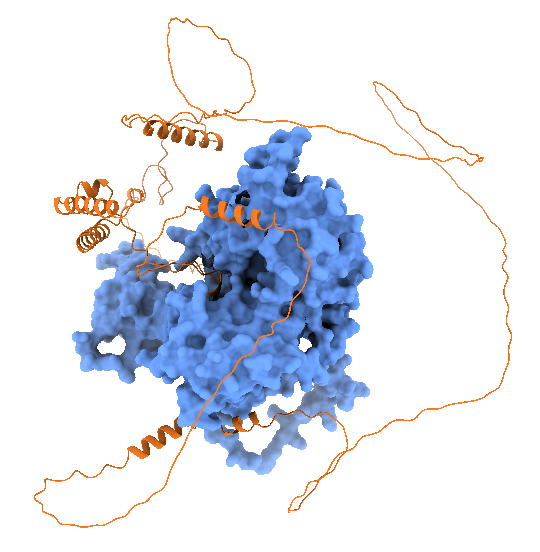

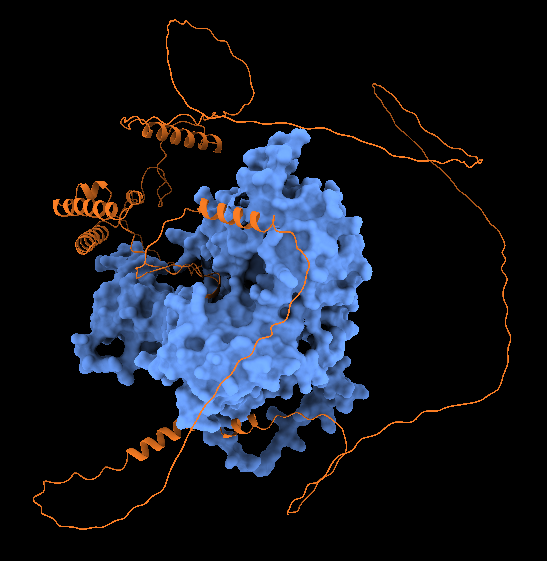


**D)** CtRecQ4 (*Chaetomium thermophilum*): N-terminus (aa 1-370)


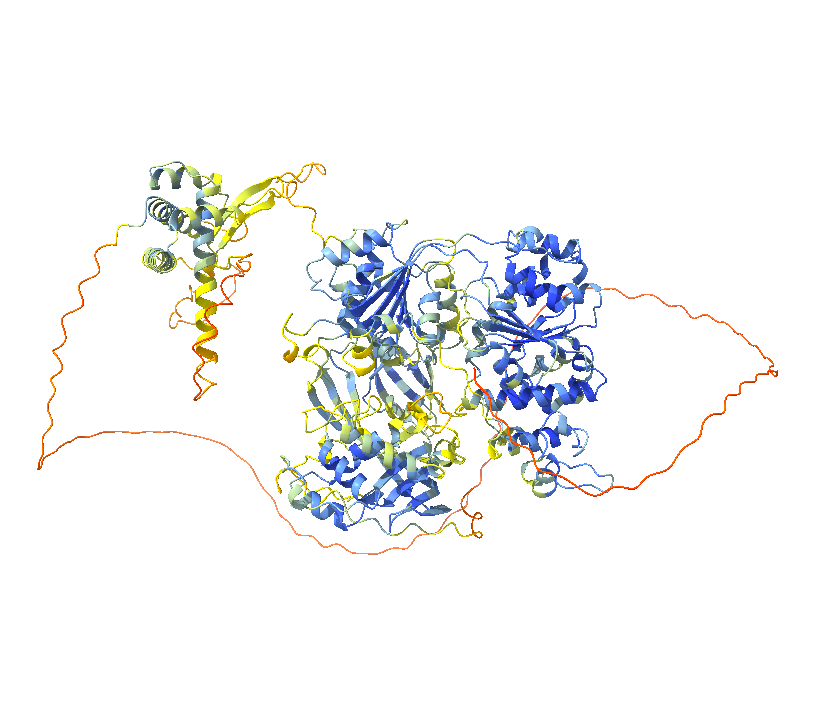

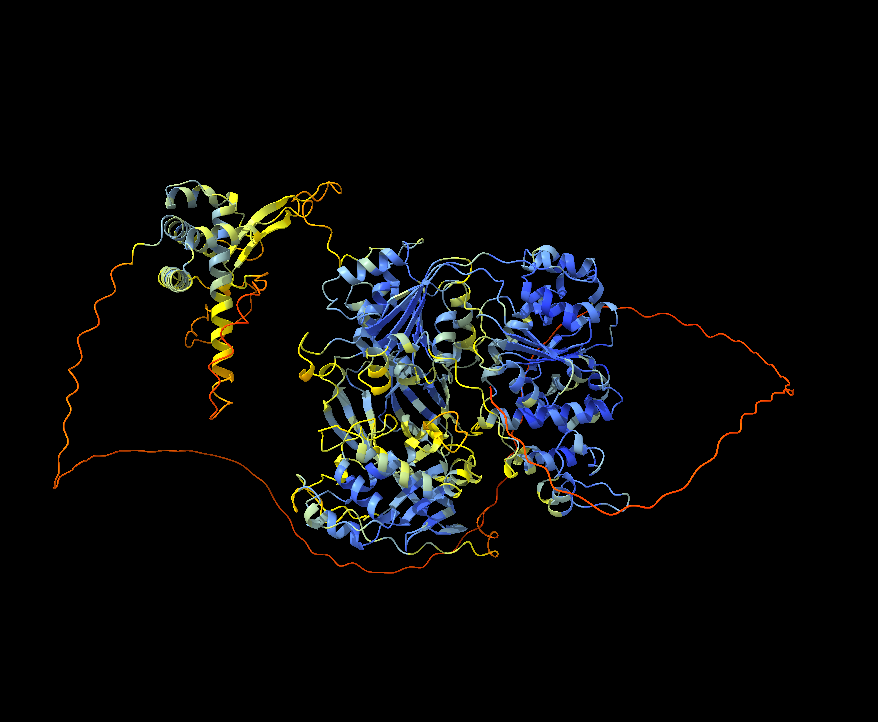

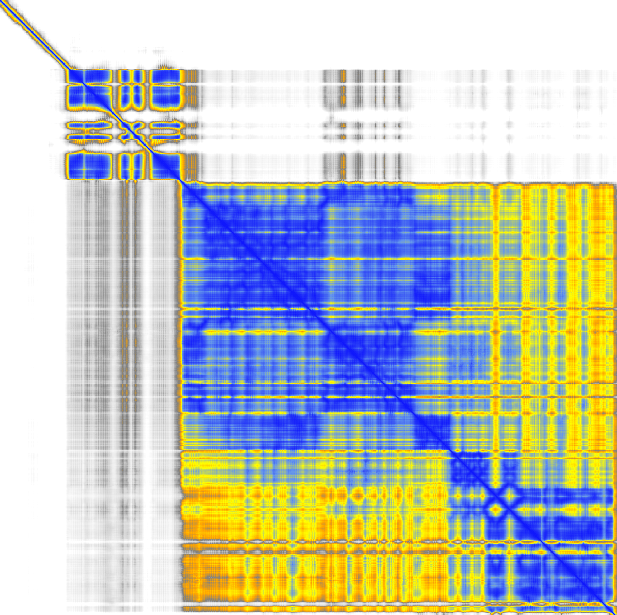


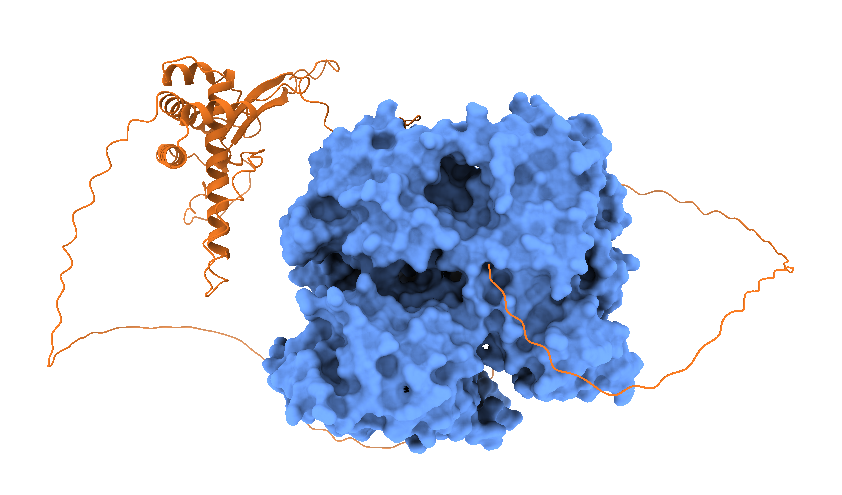

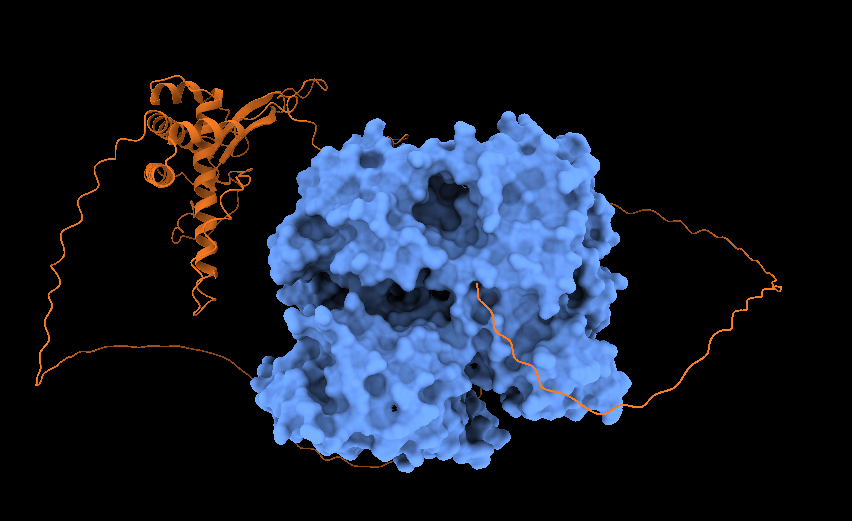


**E)** mRecQ4 (*Mus musculus*): N-terminus (aa 1-483)


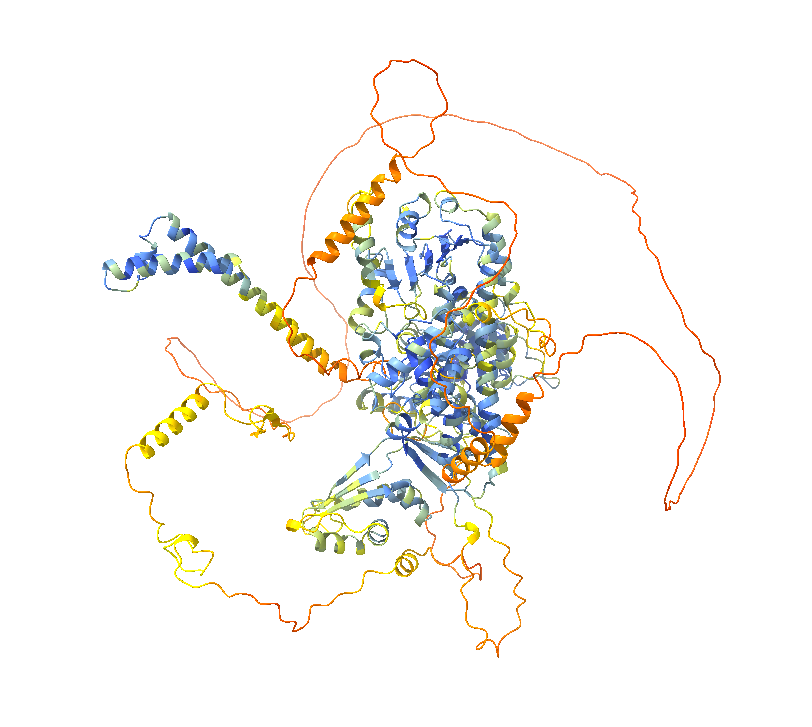

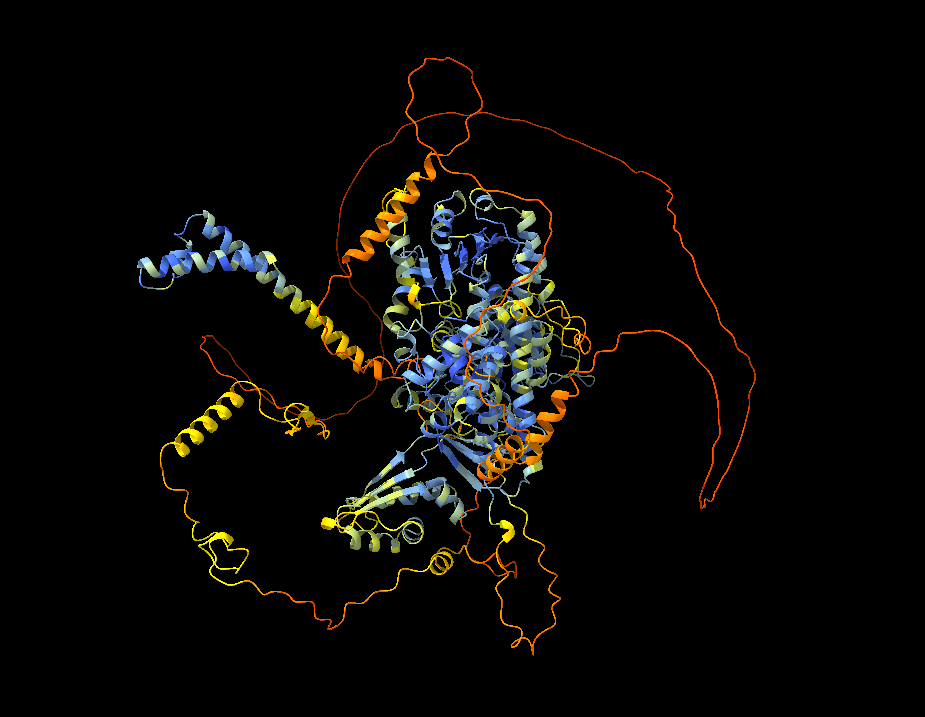

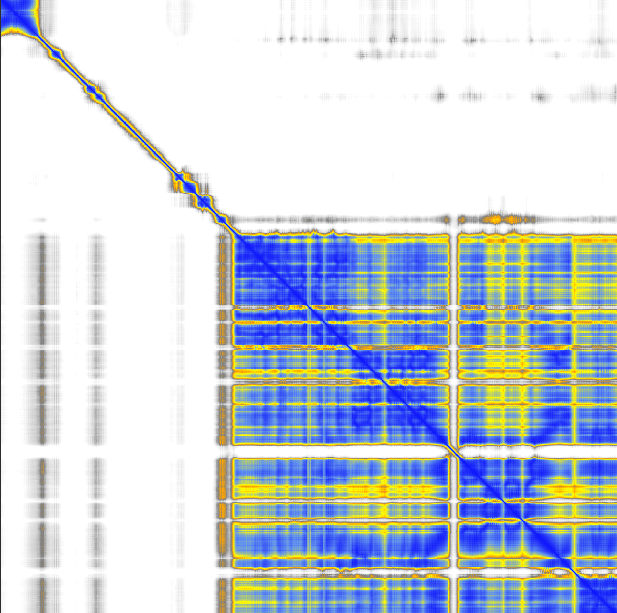


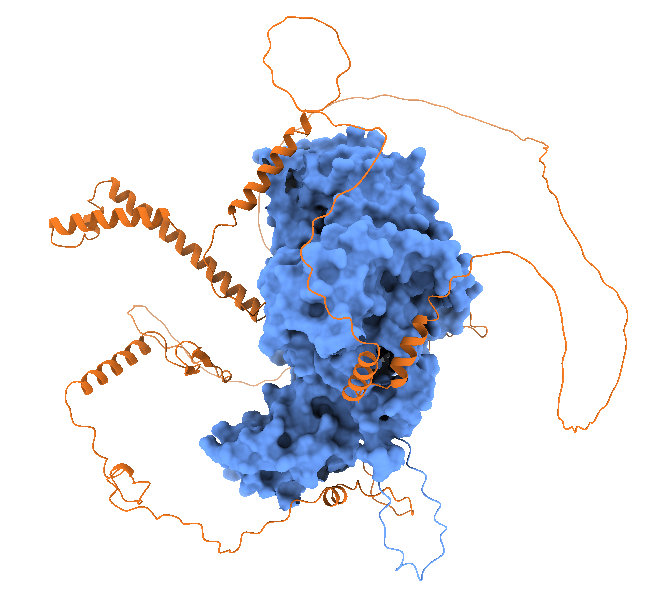

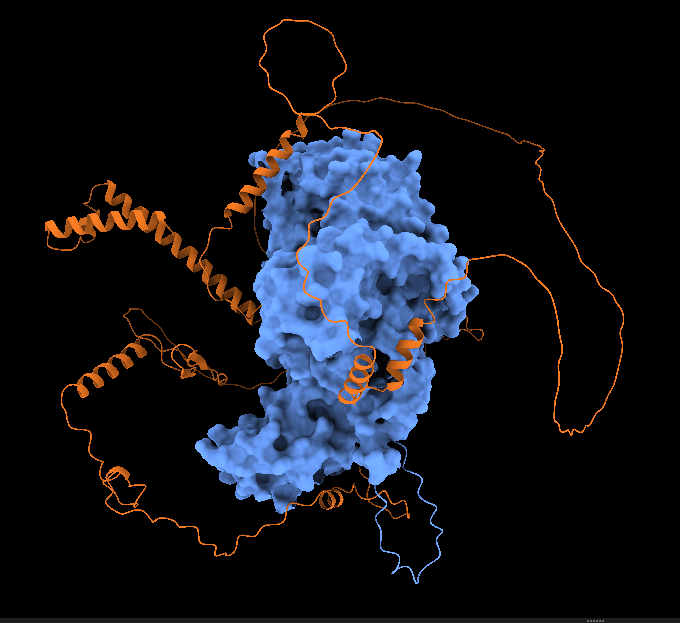


**F)** ZmRecQ4 (*Zea mays*): N-terminus (aa 1-409)


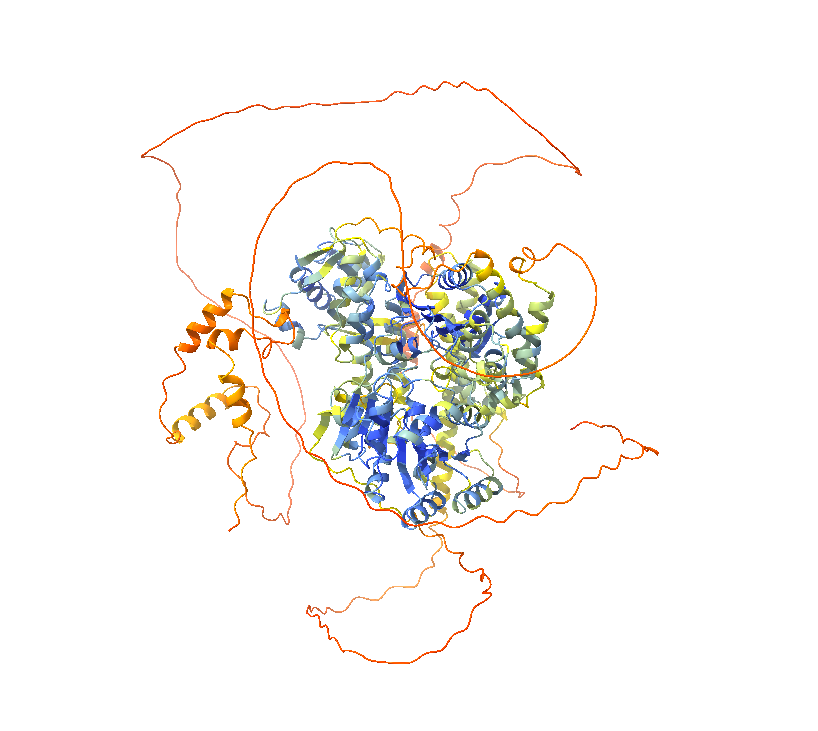

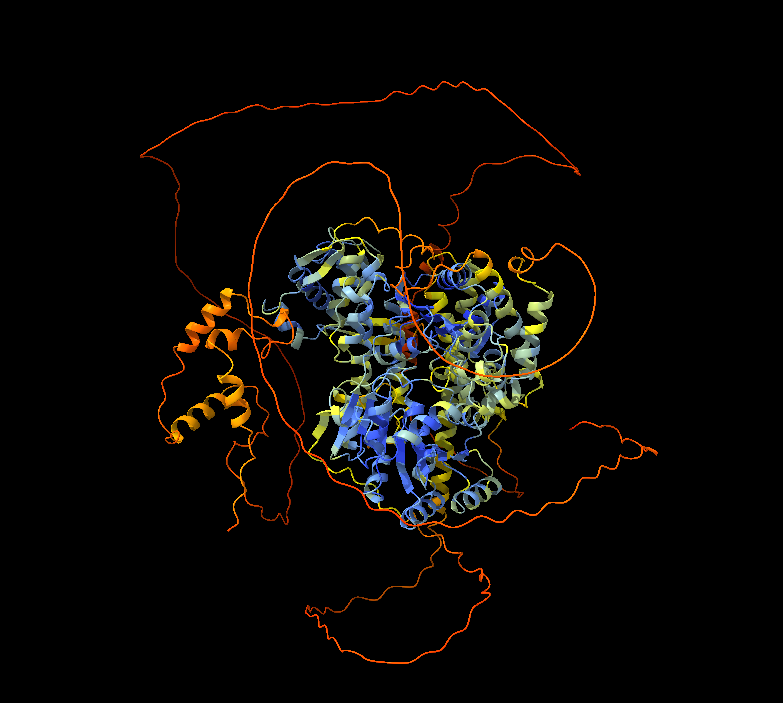

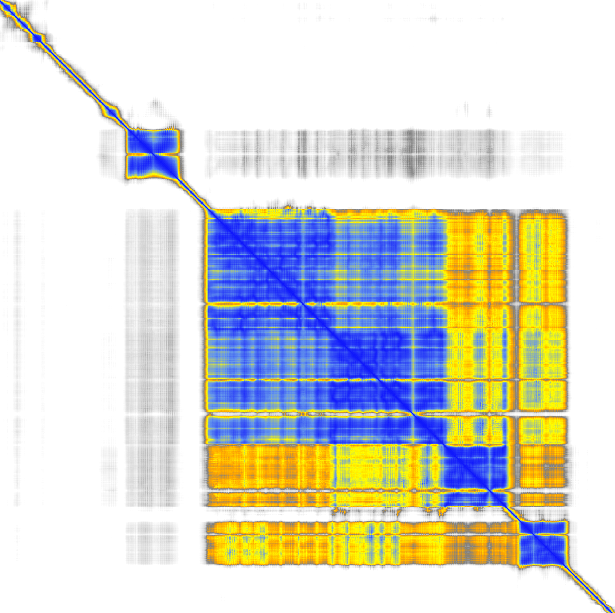


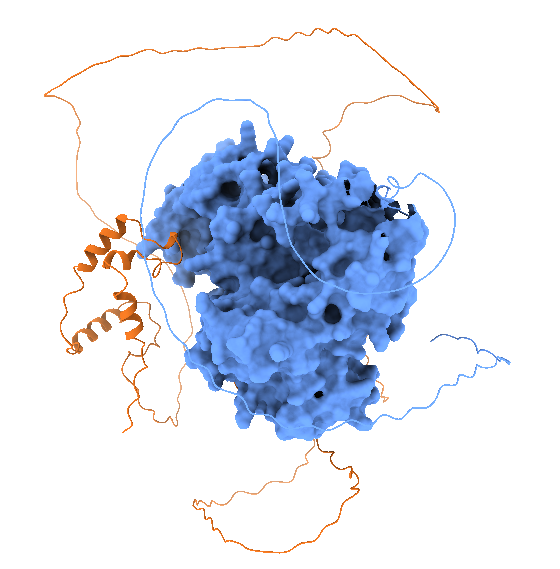

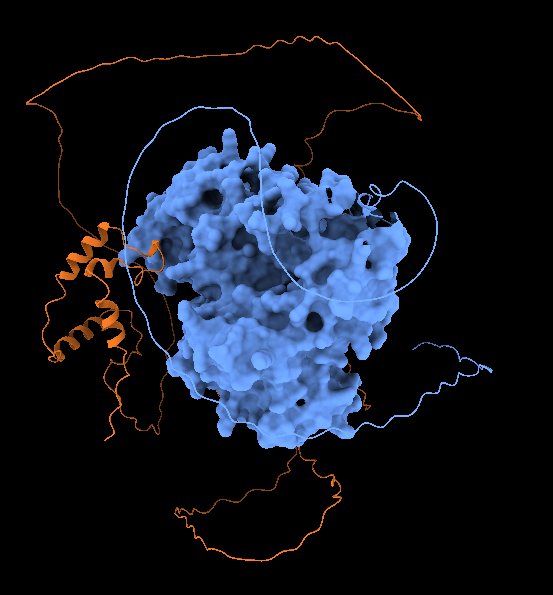


**G)** DmRecQ4 (*Drosophila melanogaster*): N-terminus (aa 1-807)


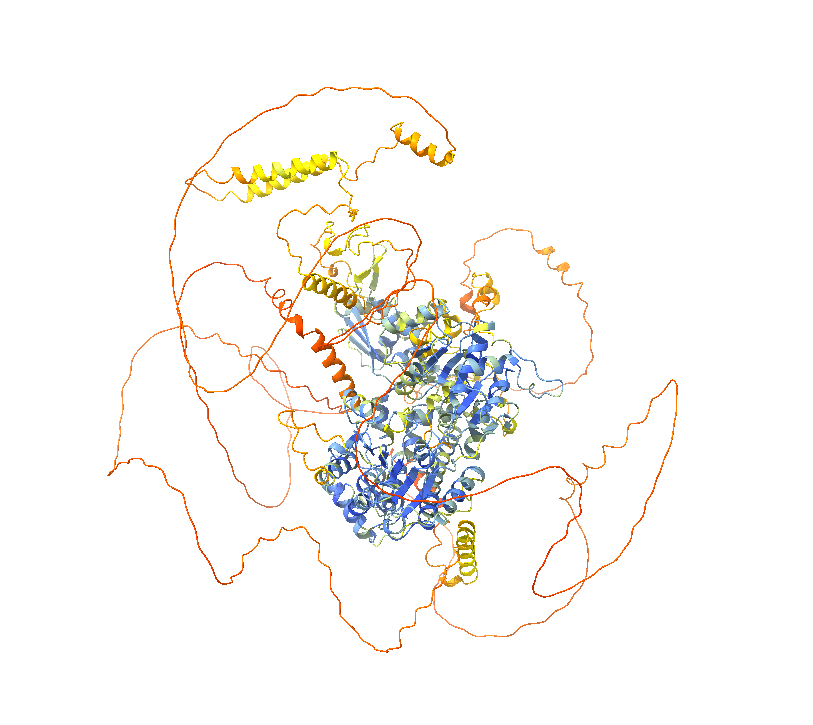

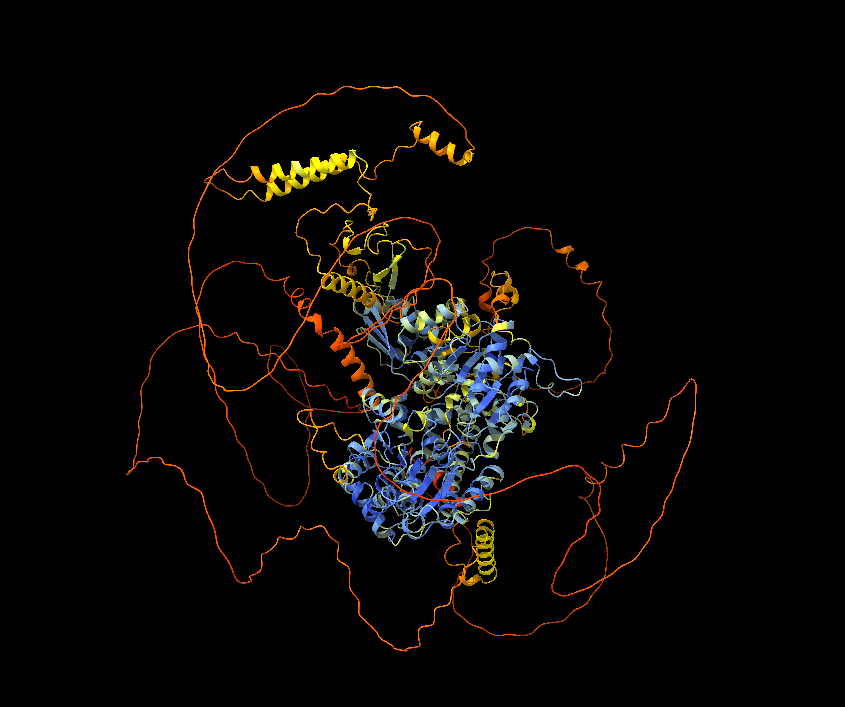

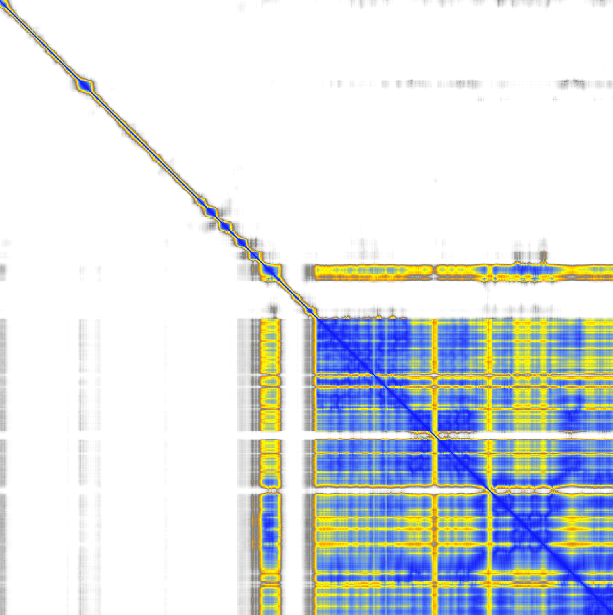


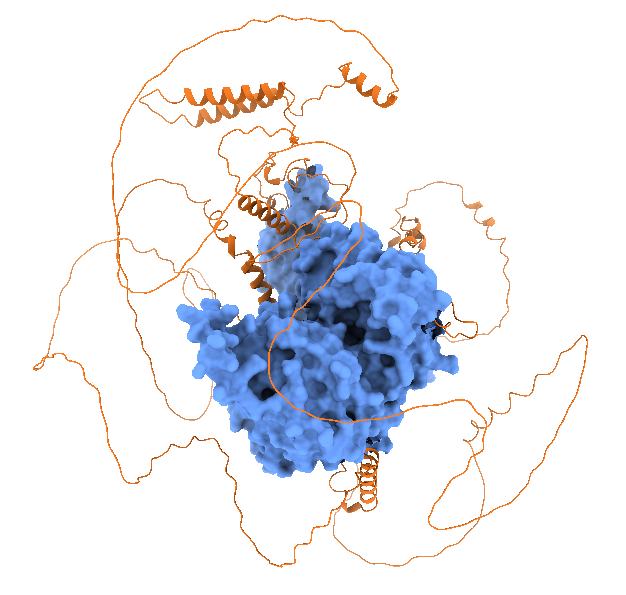

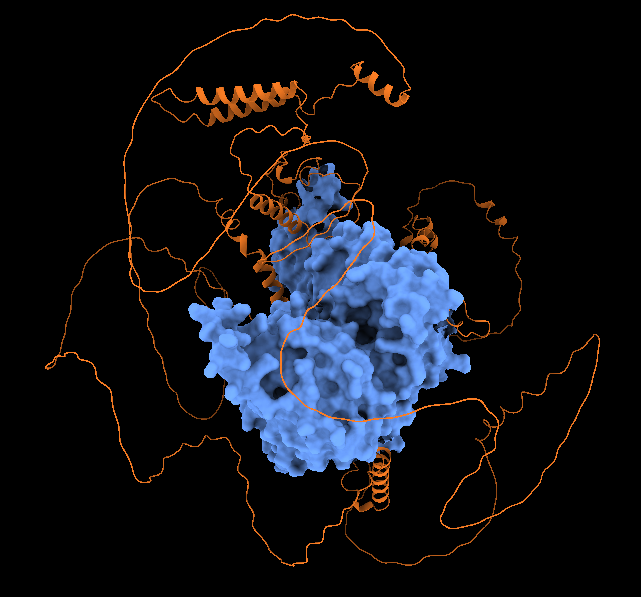


**Supplementary Figure 1. AlphaFold-predicted structures of RecQ4 family helicases.** The structures of the RecQ4 family helicases from budding yeast (A), fission yeast (B), human (C), a thermophilic fungus (D), mouse (E), corn (F), and fruit fly (G) were predicted using AlphaFold 3 and the sequences listed in Table S1. The NTDs were predicted based on the AlphaFold confidence plots and are listed for each protein. Multiple depictions of each model are included to show the confidence of the predictions and to draw attention to the NTDs *vs.* the helicase core. The structure files are included as Files S3-9.


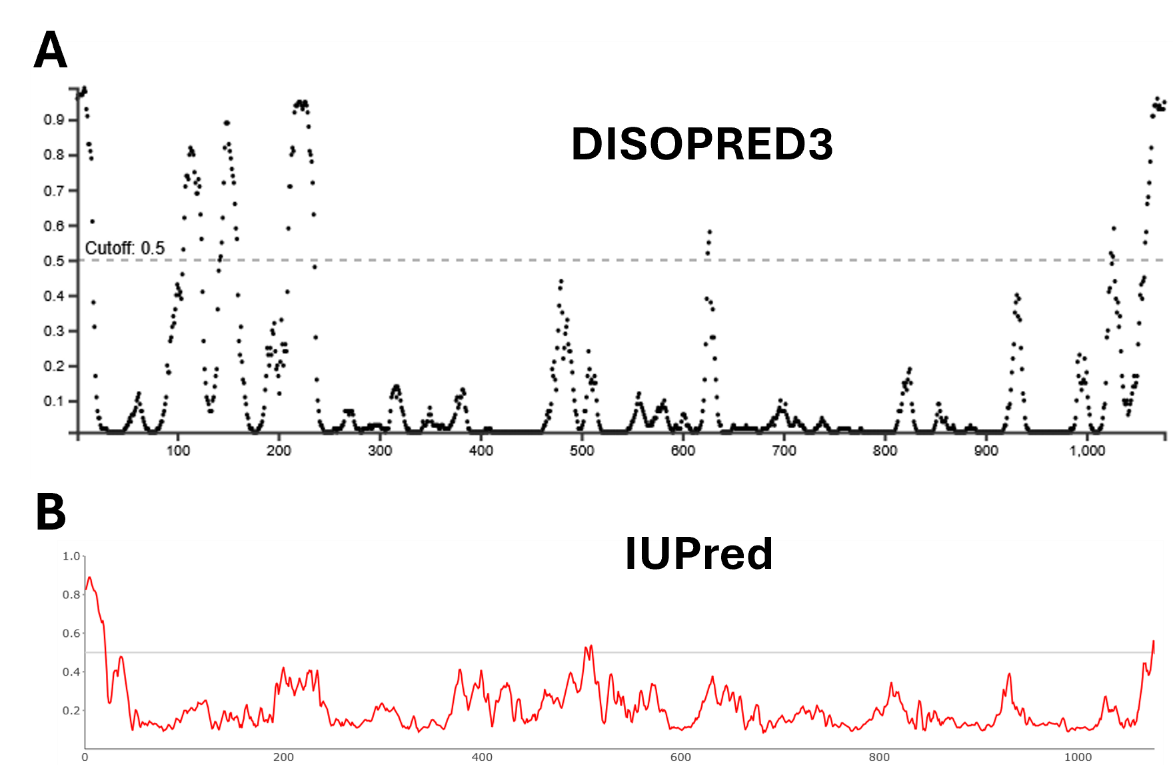


**Supplementary Figure 2. Hrq1 disorder prediction.** The DISOPRED3 **(A)** and IUPred **(B)** servers were used to analyze the *S. cerevisiae* Hrq1 sequence. Values above the 50% cutoff are considered high confidence for native disorder.

**A**


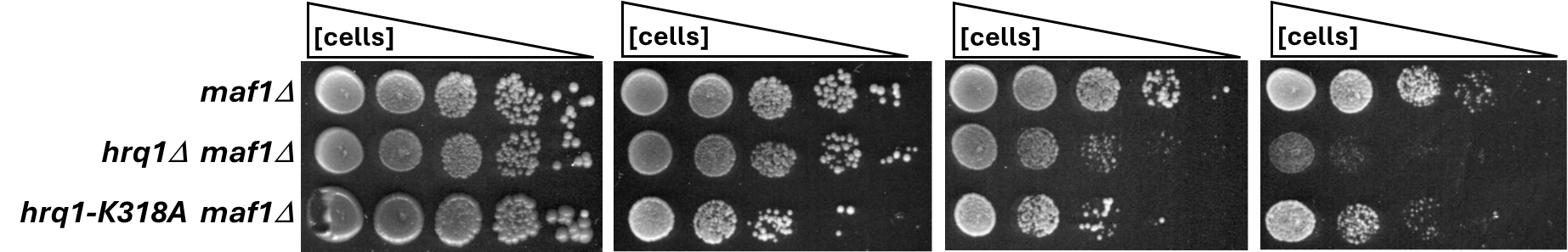


***hrq1ΔN***

***slx9Δ***

***hrq1ΔN slx9Δ***

**B**


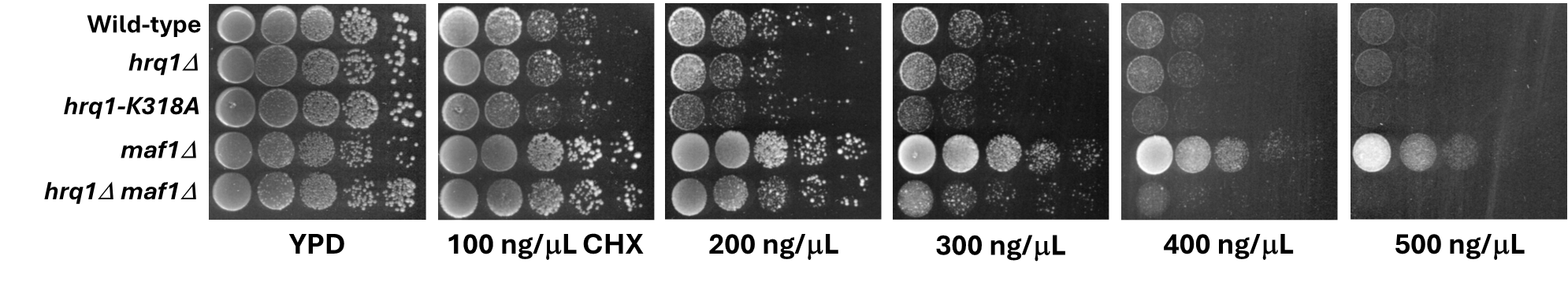

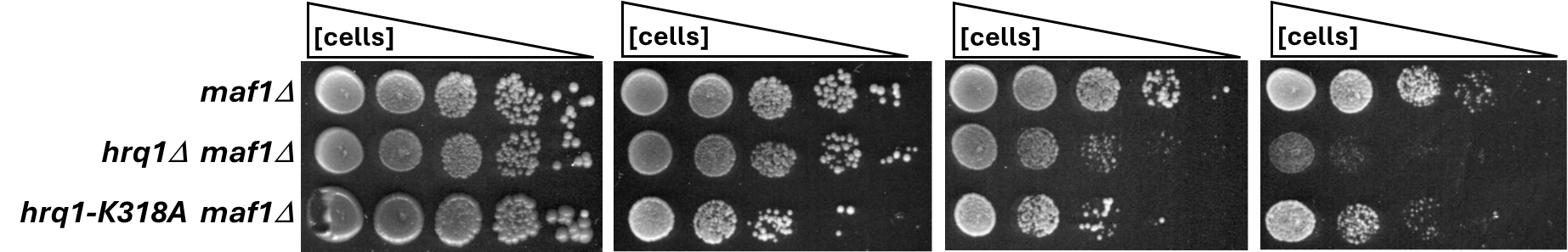


***hrq1ΔN***

***rtg2Δ***

***hrq1ΔN rtg2Δ***

**C**

**WT**

***hrq1ΔN***

***mob2-8***

***hrq1ΔN***

***mob2-8***

**Supplementary Figure 3. Confirmation of SGA hits.**  The top-five negative and positive genetic interactors from all screens were confirmed by hand. Examples are shown of a negative genetic interaction (A), a positive genetic interaction (B), and a negative genetic interaction with a temperature sensitive allele that was confirmed by growth curve analysis (C). For the spot dilution assays, overnight cultures of the indicated strains were diluted to OD_660_ = 1 and serially diluted 10-fold to 10^-4^ before plating 5 μL of each dilution on YPD. Growth curve analyses were conducted as described in (Sausen and Bochman 2021). Briefly, overnight cultures were diluted to OD_660_ = 0.01 into fresh YPD in 96-well plates, and growth was monitored for 48 h in a plate reader that was heated to 25°C or 37°C and vigorously shaking between measurements. The data were normalized to wild-type (WT), and the averages of three independent cultures are plotted; the error bars correspond to the standard deviation. *, *p* < 0.05; **, *p* < 0.01.

**SUPPLEMENTAL REFERENCES**

Sausen, C. W., and M. L. Bochman, 2021 Overcoming stochastic variations in culture variables to quantify and compare growth curve data. Bioessays 43**:** e2100108.
